# Deformation of Emulsion Droplet with Clean and Particle-Covered Interface under an Electric Field

**DOI:** 10.3390/ma13132984

**Published:** 2020-07-04

**Authors:** Muhammad Salman Abbasi, Haroon Farooq, Hassan Ali, Ali Hussain Kazim, Rabia Nazir, Aqsa Shabbir, Seongsu Cho, Ryungeun Song, Jinkee Lee

**Affiliations:** 1 Faculty of Mechanical Engineering, University of Engineering and Technology, Lahore 54890, Pakistan; hassan.ali@uet.edu.pk (H.A.); ali.h.kazim@uet.edu.pk (A.H.K.); 2 School of Mechanical Engineering, Sungkyunkwan University, Suwon, Gyeonggi-do 16419, Korea; jss1872@skku.edu (S.C.); fbsrms@skku.edu (R.S.); 3 Faculty of Electrical Engineering, University of Engineering and Technology, Lahore 54890, Pakistan; haroon.farooq@uet.edu.pk (H.F.); rabia.nazir@uet.edu.pk (R.N.); 4 Electrical Engineering Department, Lahore College for Women University, Lahore 54890, Pakistan; aqsa_shabbir@outlook.com

**Keywords:** emulsions, electric field, colloidal particles, suspensions, capsule model, colloidosomes

## Abstract

The electrohydrodynamic deformation of an emulsion droplet with a clean and particle-covered interface was explored. Here, the electrohydrodynamic deformation was numerically and experimentally demonstrated under the stimuli of moderate and strong electric fields. The numerical method involves the coupling of the Navier–Stokes equation with the level set equation of interface tracking and the governing equations of so-called leaky dielectric theory. The simulation model developed for a clean interface droplet was then extended to a capsule model for densely particle-covered droplets. The experiments were conducted using various combinations of immiscible oils and particle suspensions while the electric field strength ~10^5^ V/m was generated using a high voltage supply. The experimental images obtained by the camera were post-processed using an in-house image processing code developed on the plat-form of MATLAB software. The results show that particle-free droplets can undergo prolate (deformation in the applied electric field direction) or oblate deformation (deformation that is perpendicular to the direction of the applied electric field) of the droplet interface, whereas the low-conductivity particles can be manipulated at the emulsion interface to form a ‘belt’, ‘helmet’ or ‘cup’ morphologies. A densely particle-covered droplet may not restore to its initial spherical shape due to ‘particle jamming’ at the interface, resulting in the formation of unique droplet shapes. Densely particle-covered droplets behave like droplets covered with a thin particle sheet, a capsule. The deformation of such droplets is explored using a simulation model under a range of electric capillary numbers (i.e., the ratio of the electric stresses to the capillary stresses acting at the droplet interface). The results obtained are then compared with the theory and experimental findings. It was shown that the proposed simulation model can serve as a tool to predict the deformation/distortion of both the particle-free and the densely particle-covered droplets within the small deformation limit. We believe that this study could provide new findings for the fabrication of complex-shaped species and colloidosomes.

## 1. Introduction

A mixture of two or more immiscible liquids results in the formation of emulsions [1,2]. The electric field effects on droplets and emulsions provide a versatile avenue for exploring the underlying fluid dynamic mechanisms and instabilities [3,4,5,6,7,8,9,10,11,12,13,14,15]. It has wide applications including targeted drug delivery [16,17], particle synthesis [18], material science [19,20], electrohydrodynamic atomization [21], particle manipulation [22,23] and producing unique colloidal assemblies [24,25,26]. The major stimulating force in these processes is the electric shear stresses that are generated due to the build-up of free electric charges at the interface that produce the interfacial flows [4]. The two important parameters that govern the direction of interfacial flows are the electrical permittivity ratio (*S*) and the electrical conductivity ratio (*R*) amongst the droplet and the outside liquids.

The earliest experiments on a neutrally buoyant emulsion droplet under an electric field were conducted in 1953 [14]. Later, Taylor et al. [8] found the deformation/distortion of a particle-free leaky dielectric droplet immersed in another leaky dielectric liquid and this theory is now famously called the leaky dielectric theory. Later, experimental investigations showed that the deformation predicted by Taylor matched well in a low deformation regime but deviated at larger deformation [10]. Different authors reported that the deformation/distortion estimated by the numerical simulation showed good matching with the experimental results even at the larger deformation of the droplet [4,5,27]. Recently, it was reported that for *R* > *S,* if the degree of deformation (defined as the ratio of the stretched/deformed droplet major axis to its initial radius) exceeds a value of 1.5, the droplet becomes unstable and breaks at the ends [5]. Taylors’ theory is also invalid for the droplets with particles captured at the liquid–liquid interface. 

Colloidal particles do have a robust affinity to get adsorbed at the liquid–liquid interface and this phenomenon can stabilize the emulsions [28,29]. The electric field effects on these particles entrapped at the interface have surprisingly caught less attention, although a few intriguing reports based on the experiments attributed to the particles’ distribution and assembly at the interface can be found in the literature [24,30,31]. A low particle concentration at the interface can produce different assemblies such as the ‘belt’ of the particles around the equator or the band of the so-called ‘counter rotating vortices’ of particles [24]. Densely particle-covered droplets under a weak electric field form belts of larger width and under a strong electric field may adopt unusual ‘drum-like’ shapes [32]. The existence of particles at the interface hinders the interfacial flow, limiting it to the clean portion of the droplet [33].

Ha and Yang [34] developed a theoretical model within the small deformation limit with an aim to predict the deformation of the densely particle-covered droplets. A recent model [35] has shown that the droplet coated with particles behaves like a capsule i.e., it is bounded by a flexible (elastic) membrane. However, the theoretical models available can predict the distortion of either a particle-free or a particle-covered droplet, separately. They still are not valid for both cases, simultaneously. Therefore, a versatile model is required that is applicable to both the particle-free and the densely particle-covered droplets. 

In this article, a numerical simulation model is established as a consolidated model that can predict the low-limit deformation of both the particle-free and the densely particle-covered droplets. Here, we used COMSOL Multiphysics for the implementation of various equations governing the problem. We numerically described a particle monolayer surrounding the emulsion interface. We also performed experiments using the basic experimental facility and compared the experimental results with the theory and the simulation results. 

## 2. Materials and Methods 

### 2.1. Experimental Scheme

**Figure 1:**
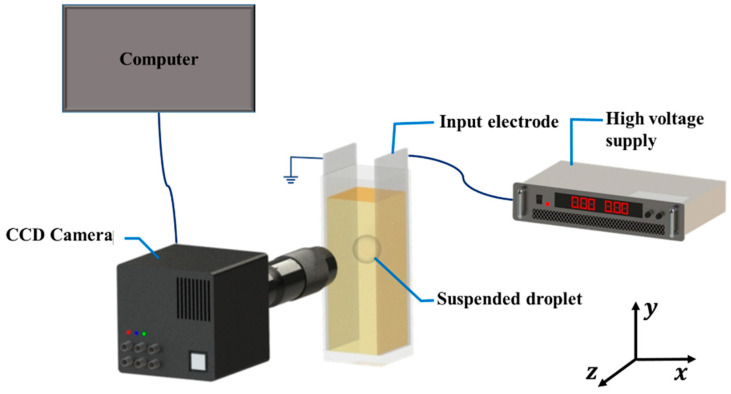
Experimental setup used for measuring the droplet distortion under an applied electric field. Charged coupled device (CCD) camera was used.

The experimental scheme for observing the droplet deformation is as shown in Figure 1 and consists of a cuvette cell (acrylic with dimensions: 2.07 cm × 2.07 cm × 5 cm) whose opposite sides were embedded with copper electrodes (0.035 cm thickness) and were filled with a leaky dielectric liquid (Liquid 1: silicone oil or castor oil). A single droplet (Liquid 2: castor oil or silicone oil) was dispensed into the cuvette using the micro-pipette. The silicone oil and the castor oil were used interchangeably as the continuous and the dispersed phases. The high DC voltage was applied by the high-voltage facility. The dynamics of the droplet under an electric field were observed using a camera and the videos were recorded at 30 frames per second (fps). The recorded videos were then post-processed to get high-quality images. An in-house code was developed on the plate-form of MATLAB (R2015a, Mathworks, Natick, MA, USA) for the image processing and the subsequent data acquisition. The obtained data were then represented in the form of the deformation (*D*) of the droplet. The physical properties of the dielectric oils used in this research are given in Table 1. The liquid systems used are summarized in Table 2.

### 2.2. Preparation of Suspension

The suspension of asphaltene particles (low-electrical conductivity) was prepared by dissolving asphaltenes in silicone oil with a concentration of 1.0 wt%. The process of particle synthesis is given in the Supplementary Information. The SEM images (See Figure S1) were used to elucidate the particle size that ranged from ~1 to ~25 μm. The detailed properties are given in Table S1. In order to dissolve the asphaltenes completely in silicone oil, it was mixed at 3200 rpm using a vortex mixer (WiseMix VM-10, DAIHAN Scientific Co. Ltd, Wonju-si, Korea). Suspension was sonicated for 30 min at room temperature to ensure the homogenous dispersion of the asphaltenes in the silicone oil. A volume of 4.2 mm^3^ of the particle suspension in the form of a droplet was dispensed into the castor oil to investigate the dynamics of asphaltene particles under an electric field. The suspensions of kaolinite clay were also formed using the same procedure.

### 2.3. Simulation

**Figure 2:**
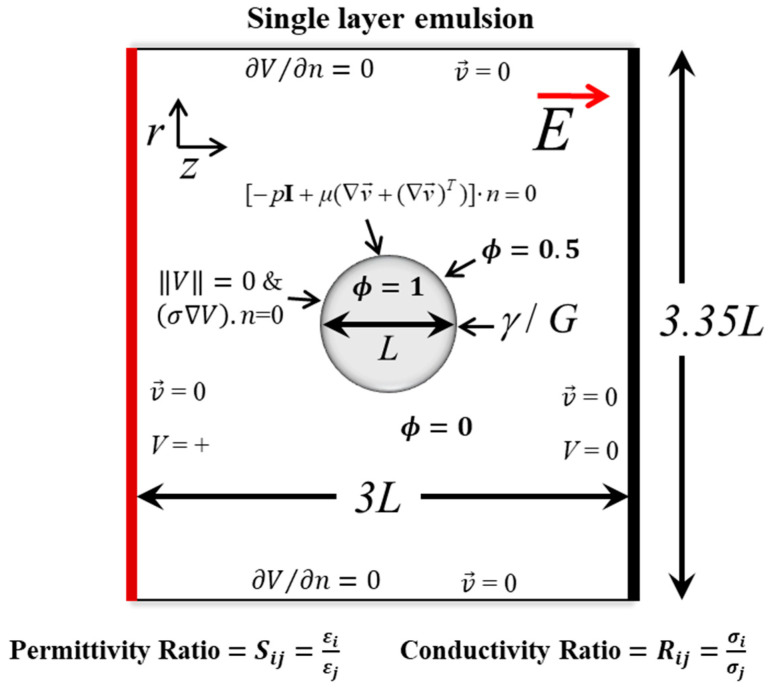
Numerical domain with the boundary conditions for studying the deformation dynamics of particle-free and particle-covered droplets subjected to an electric field $\vec{E}$. L is the droplet/suspension diameter. The voltage (V) is applied at the right side and at left side is grounded (V = 0). The top and bottom are electrically insulated (∂V/∂n=0). No slip boundary condition ($\vec{v}=0$) is applied at all the walls. The phases are represented by a value of level set function (ϕ); ϕ=0 for a continuous and ϕ=1 for a dispersed phase. At the interface, the initial fluid interface condition $\left[-pI+\mu \left(\nabla \vec{v}+{\left(\nabla \vec{v}\right)}^{T}\right)\right].n=0$ for fluid flow, and the continuity of electric potential (‖V‖=0) and the continuity of electric current [(σ∇V).n=0] for electrostatics were imposed. Here, S is the permittivity ratio, R is the conductivity ratio and γ is the interfacial tension. The subscript “i” and” j” are used to denote the liquid component at the inside and outside of the droplet, respectively. For a particle-covered droplet, γ is replaced by a shear elastic modulus, G.

By using the finite element method, the electrohydrodynamic problem under consideration was solved. Here, the Navier–Stokes equation was coupled with the equations of the electrostatics’ theory. The flow was considered to be laminar, two phase and incompressible. The numerical domain with complete boundary conditions is as shown in Figure 2. The input voltage (*V*) was applied to the right side of the domain while the left side was grounded. All the other walls were electrically insulated. No slip condition was implemented on all the walls of the domain. The initial fluid interface for fluid flow, and the continuity of electric potential and the continuity of electric current for electrostatics were imposed at the interface. 

The velocity of the flow $\;\left(\vec{v}\right)$ was calculated by the Navier–Stokes equation expressed as

(1) $$\rho \left(\frac{\partial \vec{v}}{\partial t}+\left(\vec{v}\cdot \nabla \right)\vec{v}\right)=-\nabla p+\mu \nabla^{2}\vec{v}+{\vec{f}}_{ST}+{\vec{f}}_{E}$$

(2) $$\nabla \cdot \vec{v}=0$$

where ${\vec{f}}_{E}$ is the electric force acting on the liquids obtained by coupling the fluid physics with the electrostatics physics and ${\vec{f}}_{ST}$ is the interfacial tension force between the liquids. The interface between the two liquids can be tracked by using a phase field or a level set equation [4,36,37]. In the current study, the interface was tracked by using a conservative form of the level set equation [38,39] given as

(3) $$\frac{\partial \phi }{\partial t}+\nabla \cdot (\vec{v}\phi )=\chi \nabla \cdot (\Psi \nabla \phi -\phi (1-\phi )\frac{\nabla \phi }{\left|\nabla \phi \right|})$$

where ϕ is the level set function and its value changes from ‘0’ (continuous phase) to ‘1’ (dispersed phase), Ψ is the parameter that controls the thickness of the interface, χ is the stability parameter known as the re-initialization parameter and $\phi \left(1-\phi \right)\frac{\nabla \phi }{\left|\nabla \phi \right|}$ is known as the artificial flux. The Navier–Stokes equation and the level set equation were solved using the Galerkin least square method based consistent stabilization techniques for increased stability and solution accuracy. The velocity field was solved using second-order quadratic basic functions and the pressure was solved using linear basic functions. 

The hydrodynamic properties such as density  (ρ) and viscosity  (μ) change across the interface were defined in terms of the level set function  (ϕ) as

(4) $$\rho =\rho_{2}+\phi \left(\rho_{1}-\rho_{2}\right)$$

(5) $$\mu =\mu_{2}+\phi \left(\mu_{1}-\mu_{2}\right)$$

where subscripts ‘1′ and ‘2′ refer to the respective property value at the inside and outside of the interface, respectively. 

The governing equation of the electric field for leaky dielectric fluid is given as

(6) $$\nabla \cdot \left(\sigma \vec{E}\right)=0$$

where the electric field $\vec{E}\;$ is calculated as

(7) $$\vec{E}=-\nabla \vec{V}$$

and V is voltage applied to the electrode. Second-order quadratic functions were used for calculating electric potential.

The electrical properties such as conductivity (σ) and permittivity  (ε) were also defined in terms of the level set function  (ϕ) as

(8) $$\sigma =\sigma_{2}+\phi \left(\sigma_{1}-\sigma_{2}\right)$$

(9) $$\varepsilon =\varepsilon_{2}+\phi \left(\varepsilon_{1}-\varepsilon_{2}\right)$$

The total electric force is given as

(10) $$F_{E}=\nabla \cdot \sigma_{M}$$

where $\sigma_{M}=\varepsilon \left(\vec{E}\vec{E}-E^{2}I/2\right)\;$ is the Maxwell stress tensor. Due to the action of electrical force, the interface gets distorted, resisted by the surface tension. For the numerical implementation of the deformation of densely particle-covered droplets, the interfacial tension between the liquids (γ) was re-defined in terms of the elastic shear modulus (*G*) for the particle monolayer.

We first performed the domain and grid independency tests to ensure the reliability of the simulation results based on the convergence criterion. We increased the grid resolution near the interface introducing a rectangular virtual domain using virtual operations in COMSOL Multiphysics (5.3a, COMSOL, Inc., Burlington, MA, USA). This method increased the accuracy of interface tracking without much increase in the computational loads. The final grid element size in the virtual domain and the remaining domain was set as 0.044 mm and 0.150 mm, respectively. Furthermore, the domain size of length and height equal to 3.35 L and 3 L, respectively, (L being the droplet diameter) were chosen as it avoided the effects of any domain truncation. The details about the grid independency and the domain independency tests are given in Supplementary Information (See Tables S2 and S3).

### 2.4. Theory

Leaky dielectric fluids behave differently as compared to dielectric fluids under an electric field. A small amount of charge is accumulated at the interface which is not present in perfect dielectrics. The deformation (*D)* is calculated as

(11) $$D=\frac{P-W}{P+W}$$

where *P* and *W* are the dimensions of the distorted droplet in the parallel and perpendicular directions with respect to the applied electric field. 

Here, the electric capillary number (*Ca_e_*) is defined as the fraction of electric stress to capillary stress, acting at the liquid–liquid interface of the droplet of radius *r*:

*Ca_e_* = *ε*_2_*E*_0_^2^*r*/ *γ* (12)

*ε*_2_ is the outside liquid electrical permittivity and *E_o_* is the external unperturbed electric field. *γ* is surface tension of the liquid–liquid interface. Taylor’s theory [8] for predicting the small deformation of leaky dielectric drops is:

(13) $$D=\frac{9}{16}Ca_{e}\left[\frac{R^{2}-2S+1}{{\left(2+R\right)}^{2}}+\frac{3}{5}\frac{R-S}{{\left(2+R\right)}^{2}}\left(\frac{2+3\Gamma }{1+\Gamma }\right)\right]$$

where $\Gamma =\mu_{1}/\mu_{2}$ is the viscosity ratio, $R=\sigma_{1}/\sigma_{2}$ is the conductivity ratio and $\;S=\varepsilon_{1}/\varepsilon_{2}$ is the permittivity ratio. Subscripts ‘1′ and ‘2′ represent the inlet and outlet liquids, respectively.

The particle monolayer developed at the droplet interface can be modeled as an incompressible material, called the capsule model and the deformation (*D_eq_*) is given as [33]

(14) $$D_{eq}=Ca_{s}\left[\frac{27{\left(1+R\right)}^{2}-4/S}{32{\left(2+R\right)}^{2}}\right]$$

where *Ca_s_* is the modified electric capillary number due to the existence of particles at the interface.

## 3. Results and Discussion

In this paper, the dynamics of a nearly neutrally buoyant droplet suspended in immiscible liquid subjected to an electric field is studied. A simulation model was established for understanding the deformation dynamics of the particle-free droplet, which was then extended to predict the deformation of a particle-covered droplet. The experiments were performed using silicone oil, castor oil and low-conductivity particle suspensions at the different electric capillary number ($Ca_{e}$ = *rε_o_E*_0_*^2^/γ*), which is the ratio of the electrical stresses (*ε_o_E*_0_*^2^*) countered by the capillary stresses (*γ/r*). The simulation results were then validated with the experimental findings. Owing to the slight density difference between the liquids, the resulting droplet gravitational velocity was much slower than the electrohydrodynamic flow speed. For example; for the case of the silicone droplet in castor oil, the gravitational velocity $(v_{T}\approx 2\left(\rho_{1}-\rho_{2}\right)gr^{2}/9\mu_{2})\;$ was of the order of 10^−5^ m/s and the electrohydrodynamic flow velocity was ($v_{E}\approx \;r\varepsilon_{o}E_{o}{}^{2}/\mu_{o})\;$~10^−3^ m/s. Thus, $v_{T}/v_{E}\ll 1$ where $\rho_{1}$ and $\rho_{2}$ are the densities of the dispersed and the continuous liquid, respectively, g is the gravitational acceleration, r is the droplet radius and $\mu_{2}$ is the dynamic viscosity of the continuous liquid. 

**Figure 3:**
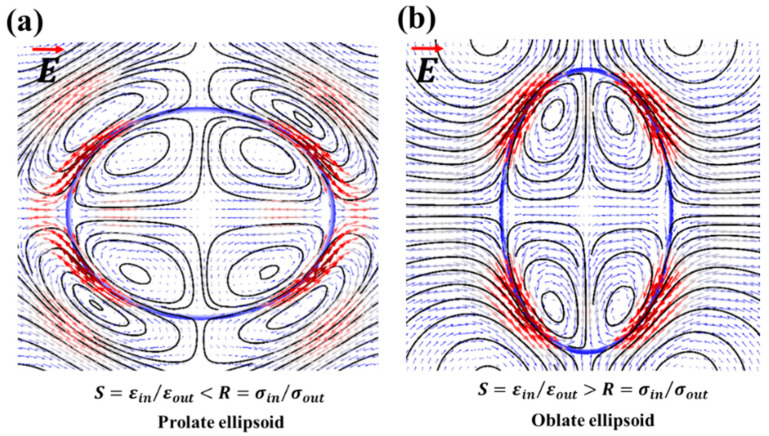
Simulation results pertaining to the distortion of the particle-free droplet under an applied electric field: (**a**) the prolate deformed droplet; the flow is from the equator towards the droplet poles; (**b**) the oblate deformed droplet; the flow is from the poles towards the droplet equator. Here, S is the permittivity ratio, whereas R is the conductivity ratio between the droplet and the ambient liquid.

A particle-free droplet was observed to show prolate (deformation that is aligned to the direction of the applied electric field) or oblate deformation (deformation that is perpendicular to the electric field direction) under the electric field. This is as shown in Figure 3. When the permittivity ratio (*S*) was less than the conductivity ratio (*R*), the upper and the lower half of the droplet developed the opposite charge of that of the input (+) and the ground electrode (−). As the opposite charge attracted, the droplet was prolate deformed and the flow was directed from the equator towards the poles. Conversely, when the *S* > *R*, the upper and the lower half of the droplet developed the same charge as that of the input (+) and the ground electrode (−), and the droplet was oblate deformed and the flow direction was now reversed i.e., from the poles towards the equator of the droplet. It should be noted that it is also possible to get prolate deformation with a flow direction from the poles towards the equator under a certain set of parametric conditions [5].

**Figure 4:**
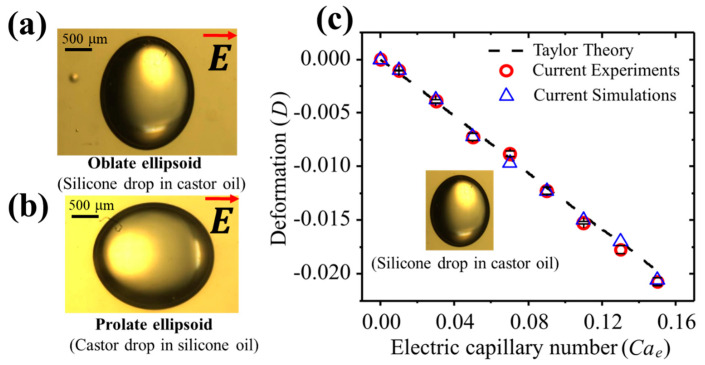
Experimental results for the particle-free droplet deformation: (**a**) the oblate deformation; SC system, (**b**) the prolate deformation; CS system, (**c**) the comparison with the simulation/experimental results and Taylor’s theory [6]. The electric capillary number was changed by increasing the electric field strength in a range from 0.025 kV/mm to 0.175 kV/mm. The radius of the droplet was fixed as 1 mm.

The experiments were performed with different immiscible liquids (refer to Table 1 and Table 2) as shown in Figure 4. It can be seen from Figure 4a,b that a silicone droplet in a castor oil shows oblate deformation, thus the flow direction would be from the poles towards the equator of the droplet whereas a castor droplet in silicone oil shows the prolate deformation with flow direction from the equator towards the poles. Here, the poles were referred to as the droplet ends nearest to the supply and ground electrodes whereas the equator ends were referred to as the droplet ends nearest to the side walls. We continued to perform experiments on the silicone droplet in castor oil under a wide range of electric capillary numbers and measured the deformation using the MATLAB image-processing tool. The results were plotted as shown in Figure 4c. Here, we changed the electric capillary number by increasing the applied unperturbed electric field strength (0.025 kV/mm to 0.175 kV/mm). The extent of the droplet deformation increased with an increase in the electric capillary number. The applied electric capillary number was always less than the threshold that can incorporate the droplet instabilities (Quincke rotation) or breakups. The experimental results were then compared with the simulation results and Taylor’s theory. A good agreement was obtained.

**Figure 5:**
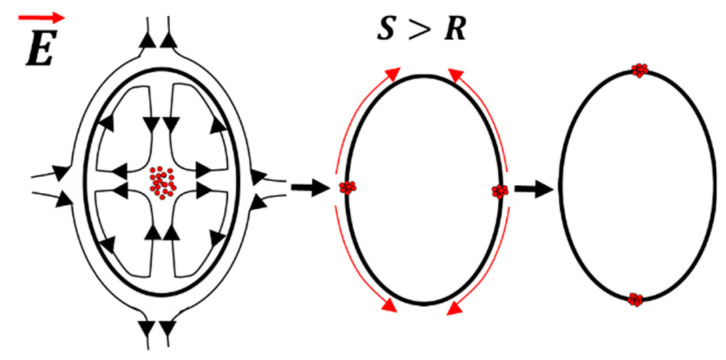
Schematic showing the transfer of particles from the suspension to the emulsion interface by electric field. Here, permittivity ratio (*S*) between the droplet and the outside liquid is greater than the conductivity ratio (*R*).

**Figure 6:**
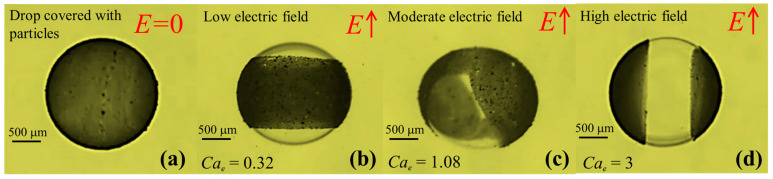
The formation of the particle-covered droplet and the effect of the electric capillary number on the particle manipulation at the interface: (**a**) the experimental image of the particle-covered droplet fabricated after using weak electric field pulses ~0.1 kV/mm^−1^ for about 2 min; (**b**) the particles moved towards the droplet equator creating a belt along it; (**c**) the particles started to migrate more towards the droplet equator ends, forming a ‘helmet’ shape; and (**d**) the particles separated into two portions, each arrested at the equator ends of the droplet in the form of ‘caps’. Here, *S* > *R*; *SC* system.

In the case of suspensions for which *S* > *R* holds, the particles were driven to the interface by applying weak electric field pulses (*E_o_* ~ 0.1 kV mm^−1^) for about 2 min. By doing so, the electrohydrodynamic flow inside the droplet pushed the particles towards the interface. These particles were then dragged by the flow at the interface towards the equator. Once entrapped at the interface, the electric field was removed. The particle distribution was then randomized at the interface and particle-covered droplets were produced. The schematic of the whole process is as shown in Figure 5, while the experimental image of a produced particle-covered droplet is shown in Figure 6a. It was found that suspensions for which *S* < *R,* the particles seemed unaffected by the electric field and did not move towards the emulsion interface. Particle interfacial dynamics under weak electric fields are as shown in Figure 6b. In case of the oblate deformed droplets, particles moved towards the droplet equator creating a ‘belt’ along it. The portions of the interface near the droplet equator became particle free. As the electric field is increased, the thickness of the belt is further decreased, and the particles start to migrate towards the droplet equator ends, shrinking the belt width along the equator. This is as shown in Figure 6c. The resulting interfacial structure resembles a “helmet” with an opening on both sides of the droplet. Under strong electric field (See Figure 6d), the oblate deformed droplet and particles were separated into two portions, each arrested at the equator ends of the droplet in the form of “caps”. These “particle caps” remained stable, however, if the electric field was removed, the caps elongated over the droplet interface and moved towards one another. Eventually, they met and the whole droplet interface was again blanketed by particles (Figure S2).

**Figure 7:**
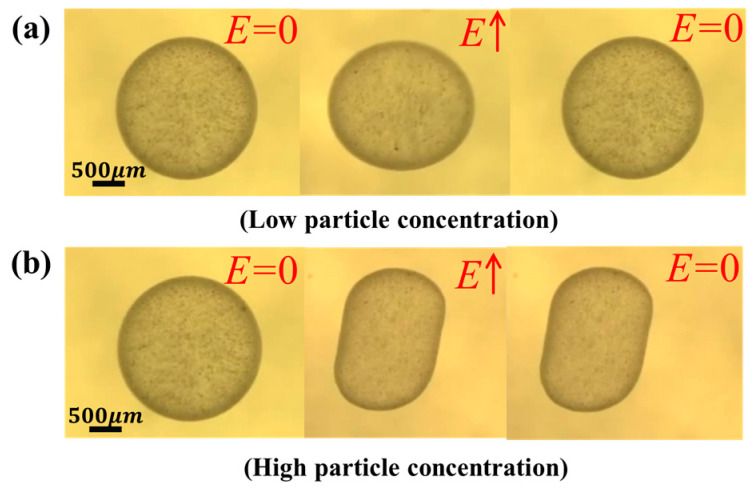
Experimental results showing the formation of non-spherical colloidosomes using an electric field: (**a**) the low particle concentration results in restoring the spherical shape after the electric field is removed. Electric field strength ~0.15 kV/mm^−1^; (**b**) the high particle concentration results in particle jamming at the interface and the spherical shape is not restored. Electric field strength ~0.35 kV/mm^−1^ and the drop radius is 1 mm.

It is pertinent to mention here that though the particle-free droplets regain their spherical shape (low surface energy configuration) after the removal of the electric field, the particle-covered droplets may or may not. Experiments were also performed with kaolinite clay suspensions as shown in Figure 7. The clay particles of different concentrations were dissolved in silicone oil to make the suspension and was suspended in castor oil. When the particle concentration was low, the droplet regained its spherical shape (Figure 7a). On the contrary, when a very large concentration of clay particles in the emulsion suspension was used, then the action of electric field caused particle jamming at the interface, resulting in the formation of stable non-spherical colloidosomes that do not regain their initial shape (Figure 7b).

As far as the deformation aspects of the densely particle-covered droplets is concerned, it was reported by Ouriemi et al. [33] that if a particle-covered droplet or an armoured droplet with *S >R* was subjected to an electric field; it behaves differently compared with a particle-free droplet. Here, only the droplets with high particle concentrations/coverage were considered. For such droplets, a mono layer of closely packed particles covered the entire interface. It behaved like an elastic sheet capable of supporting and withstanding the anisotropic stresses and strains [40]. This solid-like behavior of the interface can be characterized in terms of solid properties such as Young’s modulus and Poisson’s ratio [41,42]. Such capsules deformed more compared to the droplets with clean interfaces under an electric field and may even buckle under strong uniaxial compressive or even crack under strong tensile electric stresses. This was due to the interface/surface modification owing to the entrapped particles. The deformation of such droplets can no longer be predicted by the Taylors theory or by treating the emulsion interface as a liquid–liquid entity. Therefore, the solid-like interface behaviour was incorporated in our simulation model in terms of a modified electric capillary number (*Ca_s_*) defined in terms of a solid property such as

(15) $$Ca_{s}=\frac{\varepsilon_{2}E_{o}^{2}r}{G}=Ca_{e}\frac{\gamma }{G}$$

where a modified electric capillary number (*Ca_s_*) is defined as the ratio of electric stresses to the shear elastic stresses, and *G* is the elastic shear modulus. The value of *G* for the densely packed particle at the emulsion interface can be taken as 1.43 times the interfacial tension (γ) for a particle-free interface [40]. 

**Figure 8:**
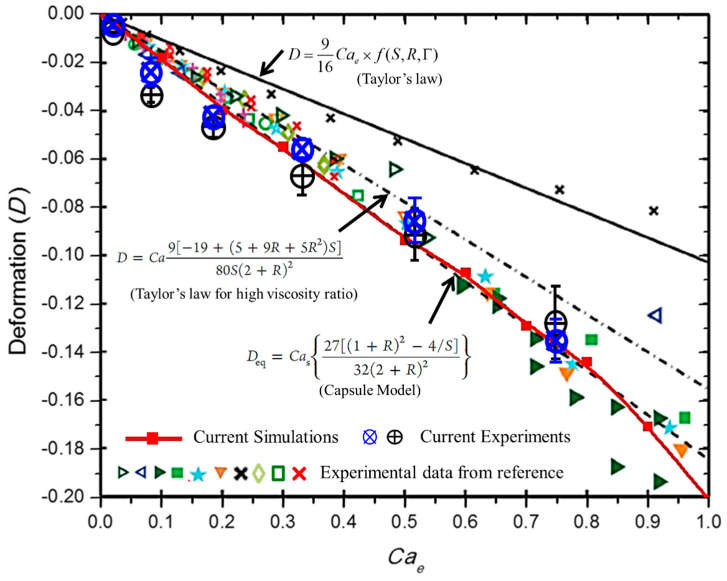
Simulation and experimental results pertain to the deformation of a particle-covered droplet under an electric field. For the experiments, we used two different asphaltene particle suspensions in silicone oil produced from asphaltene particles synthesized from two different feed stocks. The suspensions were dispensed in castor oil and the deformation of the particle-covered droplet was measured. The red line with filled rectangular symbols is obtained from our simulation model. The results are also compared against the experimental findings of Ouriemi et al. [33] for various types of particles, indicated by the different symbols and the capsule model (Equation (14)). The black solid, dot dashed, and dashed lines represent the theoretical models.

We performed extensive simulations to predict the deformation of the particle-covered droplet using a simulation model at a different electric capillary number and the results are plotted in Figure 8. The simulation results were compared with our experimental results. Furthermore, the results were also compared with a capsule model and the experimental results for the densely particle-covered droplet from another researcher [33]. A good agreement can be observed between our simulation model, the experimental results and the capsule model. Thus, our simulation model predicted the deformation for both cases effectively.

## 4. Conclusions

In this article, the electrohydrodynamic deformation of a nearly neutrally buoyant droplet with a clean and particle-covered interface were studied. The main novelty of the current work lied in implementing the simulation model as a consolidated model to predict the deformation of both the particle-free and the densely particle-covered droplets. First of all, we built a simulation model by coupling Navier–Stokes equation of fluid flow with governing equations of electrostatics theory. The interface was tracked by a conservative level set function. We tested the model against the theory and our experiments for particle-free droplets under varying conditions. The experiments were performed using a cuvette cell and an electric field was generated. Deformation was measured by an in-house code developed using the MATLAB image-processing tool. We then extracted the low conductivity asphaltene particles from a raw feedstock and observed their behavior at the droplet interface. The permittivity ratio (*S*) and conductivity ratio (*R*) between the droplet and the outside liquid emerged as two important parameters deciding the direction of the droplet distortions, the direction of the flow patterns and/or the possibility of the fabrication of particle-covered droplets. Weak electric field pulses were used to move the particles to the emulsion interface. Depending on the particle concentrations, stable non-spherical colloidosomes can be formed. For predicting the deformation of the particle-covered droplet, we adopted a simplified approach of re-defining the interfacial tension (*γ*) in the simulation model in terms of the elastic shear modulus (*G*) of the particle mono layer. The experimental results for the particle-covered droplets were then compared with the theory and our simulation results. Our simulation model emerged as a consolidated model and effectively predicted the deformation for both the cases. Thus, it can be used as a tool to predict the deformation for both cases within the small deformation limit i.e., when the droplet instabilities such as breakups or electrorotation (Quincke rotation) are not produced.

We expect that the developed model and experimental findings contribute to provide a guideline to control the emulsion droplet shape and particle assembly. If the current oils are replaced with UV-cured polymer solutions, the complex shape of the species or colloidosomes can be fabricated. 

## Figures and Tables

**Table 1:** Physical properties of the tested base liquids [5].

Liquid	Mass Density (kg/m^3^)	Dynamic Viscosity (Pa.s)	Electrical Conductivity (S/m)	Dielectric Constant
Castor Oil	961	0.78	3 × 10^−11^	4.7
Silicone Oil	970	0.97	0.87 × 10^−13^	3.2

**Table 2:** Liquid systems used in the study.

System	Dispersed Phase	Continuous Phase	Conductivity Ratio (*R*)	Permittivity Ratio (*S*)	Viscosity Ratio (Γ)
*CS*	Castor oil	Silicone oil	34.48	1.47	0.804
*SC*	Silicone oil	Castor oil	0.68	0.029	1.24

## Supplementary Materials

The following are available online at https://www.mdpi.com/1996-1944/13/13/2984/s1, Figure S1: SEM images of the asphaltenes particles; (A) at a scale of 100 μm, (B) at a scale of 10 μm. Figure S2: The caps elongate over the drop interface and cover the entire interface as the electric field is removed. Table S1: Properties of the asphaltene particles used in experiments. Table S2: Systems for grid independence tests. Table S3: Grid independency and domain independency tests.
