# Supplementary material for: Deformation of Emulsion Droplet with Clean and Particle-Covered Interface under an Electric Field
Source: Materials (Basel). 2020 Jul 4;13(13):2984. doi: 10.3390/ma13132984 (PMC7372396; doi:10.3390/ma13132984)
Supplement: Supplementary file 1 [file materials-13-02984-s001.pdf]

materials

Supplementary Materials

Deformation of Emulsion Droplet with Clean and Particle-Covered Interface under an Electric Field

Muhammad Salman Abbasi [1,2,*], Haroon Farooq [3], Hassan Ali [1], Ali Hussain Kazim [1], Rabia Nazir [3], Aqsa Shabbir [4], Seongsu Cho [2], Ryungeun Song [2] and Jinkee Lee [2,*]

[1] Faculty of Mechanical Engineering, University of Engineering and Technology, Lahore 54890, Pakistan; hassan.ali@uet.edu.pk (H.A.); ali.h.kazim@uet.edu.pk (A.H.K.)
[2] School of Mechanical Engineering, Sungkyunkwan University, Suwon, Gyeonggi-do 16419, Republic of Korea;
jss1872@skku.edu (S.C.); fbsrms@skku.edu (R.S.)
[3] Faculty of Electrical Engineering, University of Engineering and Technology, Lahore 54890, Pakistan; haroon.farooq@uet.edu.pk (H.F.); rabia.nazir@uet.edu.pk (R.N.)
[4] Electrical Engineering Department, Lahore College for Women University, Lahore 54890, Pakistan; aqsa_shabbir@outlook.com
* Correspondence: m.salman@uet.edu.pk (M.S.A.); lee.jinkee@skku.edu (J.L.)

Received: 11 May 2020; Accepted: 29 June 2020; Published: 4 July 2020

Synthesis of Particles:

Asphaltenes, the heaviest fraction of unconventional crude oils, were separated by using n-heptane as a precipitation solvent at room temperature. Three unconventional crude oils raw feed stocks, namely Laguna (Venezuela), Bachaquero-13 (Venezuela) and Rubiales (Colombia), were mixed with n-heptane at a solvent to crude oil ratio of 25 ml/g. The crude oils and n-heptane mixture was mixed gently in a shaking incubator (Lab Companion Model # IST-3075R) at 250 rpm for 6 hours. After ensuring the sufficient mixing, the mixture was placed for 4 hours to settle the precipitated asphaltenes, which were then filtered with Whatman filter paper No. 5. The n-heptane soluble fractions (saturates, aromatic and resins) were filtered from the filter paper by leaving a filter cake of precipitated asphaltenes. Then, the filter paper was dried in the drying oven at 120 °C overnight for the complete removal of n-heptane.

The dried filter cake was further extracted using toluene by Soxhlet extraction at 150 °C for 48 hours until the extracting solvent become colorless. The toluene was then dried in vacuum evaporation at 90 °C and −0.08 MPa vacuum pressure for 1 hour. After the complete removal of toluene, the solid asphaltenes were again dried in the drying oven at 120 °C until their weight became constant. The particle size of the dried solid asphaltenes was reduced for homogeneity purposes by ball milling (Pulverisette 23 Mini mill, FRITSCH, Germany) for 15 min. The dried asphaltenes in fine powder form were further used for all the experiments.

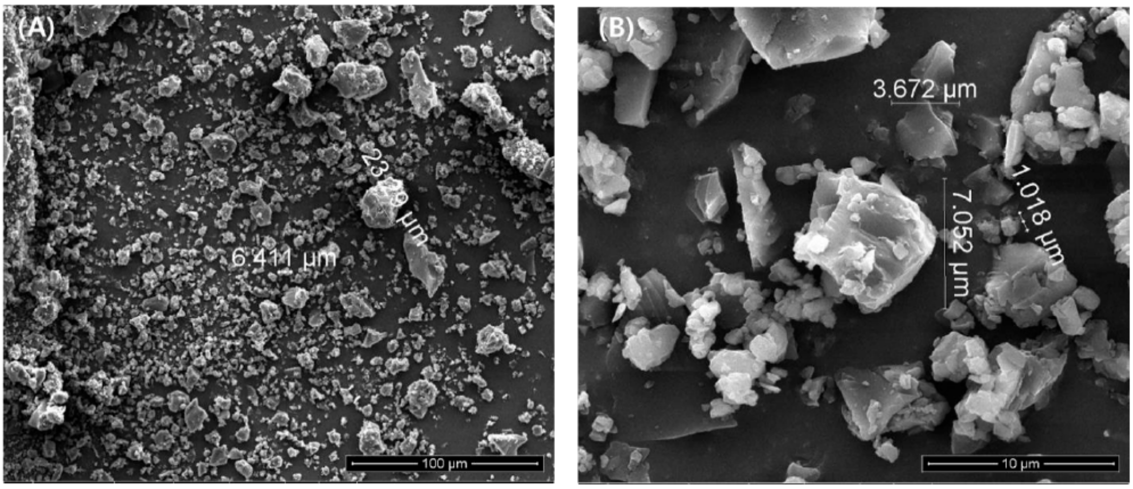

Figure S1. SEM images of the asphaltenes particles (**A**) at a scale of 100 µm and (**B**) at a scale of 10 µm.

Table S1. Properties of asphaltene particles used in experiments.

Particle Size	Dielectric Constant	Electrical Conductivity (S/m)
~1 to ~25 µm	4.3–5.4 [1]	10^{-11}–10^{-12} [2]

Grid and Domain Independency Tests:

We introduced a rectangular virtual domain to increase the grid resolution near the interface. Three different grid size combinations, each, were used in virtual and remaining domain. The details are summarized in Table S2.

Table S2. Systems for grid independence tests.

Grid Size	Grid 1 Element Size [mm]	Grid 2 Element Size [mm]	Grid 3 Element Size [mm]
Virtual Domain	47×10^{-3} L (0.094)	22×10^{-3} L (0.044)	11×10^{-3} L (0.022)
Remaining	75×10^{-3} L (0.15)	75×10^{-3} L (0.15)	75×10^{-3} L (0.15)

We measured the deformation (*D*) of the droplet by varying the electric capillary number for each of the grid setting (See. Table S3). As it can be seen that the obtained deformation doesn't change by further decreasing the grid size beyond 0.044 mm in virtual domain (Grid 2), this grid size was chosen for all the simulations to avoid any unnecessary computational loads. Further we, checked the domain effects by increasing domain length from 3.35 L (Domain 1) to 4 L (Domain 2). As can be seen from Table S3, it doesn't affect the results and thus the Domain I was chosen for the simulations.

Table S3. Grid independency and domain independency tests.

		**Simulations *D* (×10^{-3})**
$Ca_e = 0.1$ ($S = 0.1$, $R = 2.5$, $\Gamma = 1$)	Grid 1	65
	Grid 2	29
	Grid 3	29
		**Simulations *D* (×10^{-3})**
$Ca_e = 0.1$ ($S = 0.1$, $R = 2.5$, $\Gamma = 1$)	Domain 1	29
	Domain 2	29

Figure. S2 shows the two caps that elongate as the electric field is removed and then cover the entire interface.

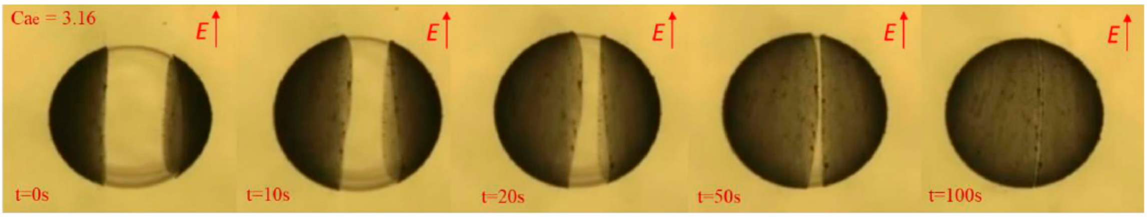

Figure S2. The caps elongate over the drop interface and cover the entire interface as the electric field is removed.

References

1. Evdokimov, I.N.; A.P. Losev. Electrical conductivity and dielectric properties of solid asphaltenes. *Energy and fuels* 2010, *24*, 3959–3969.
2. Zeng, H., et al., Critical nanoaggregate concentration of asphaltenes by direct-current (DC) electrical conductivity. *Energy and fuels* 2009, *23*, 1201–1208.

© 2020 by the authors. Licensee MDPI, Basel, Switzerland. This article is an open access article distributed under the terms and conditions of the Creative Commons Attribution (CC BY) license (http://creativecommons.org/licenses/by/4.0/).
